# Intravital microscopic optical coherence tomography imaging to assess mucus-mobilizing interventions for muco-obstructive lung disease in mice

**DOI:** 10.1152/ajplung.00287.2019

**Published:** 2020-01-29

**Authors:** Mario Pieper, Hinnerk Schulz-Hildebrandt, Marcus A. Mall, Gereon Hüttmann, Peter König

**Affiliations:** ^1^Institute of Anatomy, University of Lübeck, Lübeck, Germany; ^2^Airway Research Center North, German Center for Lung Research, Lübeck, Germany; ^3^Institute of Biomedical Optics, University of Lübeck, Lübeck, Germany; ^4^Department of Translational Pulmonology, Translational Lung Research Center Heidelberg, German Center for Lung Research, University of Heidelberg, Heidelberg, Germany; ^5^Department of Pediatric Pulmonology, Immunology and Intensive Care Medicine, Charité-Universitätsmedizin Berlin, Berlin, Germany; ^6^Berlin Institute of Health, Berlin, Germany

**Keywords:** intravital imaging, muco-obstructive disease, mucus, mucus-mobilizing therapy, optical coherence tomography

## Abstract

Airway mucus obstruction is a hallmark of chronic lung diseases such as cystic fibrosis, asthma, and COPD, and the development of more effective mucus-mobilizing therapies remains an important unmet need for patients with these muco-obstructive lung diseases. However, methods for sensitive visualization and quantitative assessment of immediate effects of therapeutic interventions on mucus clearance in vivo are lacking. In this study, we determined whether newly developed high-speed microscopic optical coherence tomography (mOCT) is sensitive to detect and compare in vivo effects of inhaled isotonic saline, hypertonic saline, and bicarbonate on mucus mobilization and clearance in *Scnn1b*-transgenic mice with muco-obstructive lung disease. In vivo mOCT imaging showed that inhaled isotonic saline-induced rapid mobilization of mucus that was mainly transported as chunks from the lower airways of *Scnn1b*-transgenic mice. Hypertonic saline mobilized a significantly greater amount of mucus that showed a more uniform distribution compared with isotonic saline. The addition of bicarbonate-to-isotonic saline had no effect on mucus mobilization, but also led to a more uniform mucus layer compared with treatment with isotonic saline alone. mOCT can detect differences in response to mucus-mobilizing interventions in vivo, and may thus support the development of more effective therapies for patients with muco-obstructive lung diseases.

## INTRODUCTION

Impaired mucus clearance leading to airway mucus obstruction is a key abnormality in cystic fibrosis (CF), severe asthma, and chronic obstructive pulmonary disease (COPD) (9, 10, 12, 13), and removal of excess mucus is an important therapeutic strategy for these muco-obstructive lung diseases. Mucociliary clearance from the lower airways to the larynx is mediated by ciliary beating on airway epithelial surfaces, cough, and airflow generated by breathing (7, 24, 30). In health, these mechanisms work together to prevent mucus accumulation and plugging of the airways. In muco-obstructive lung diseases, impaired mucociliary clearance leads to mucus accumulation in the airways requiring therapeutic intervention to remove excess mucus (9, 44). This complex process cannot be easily modeled in vitro or ex vivo (32), and methods are warranted to study airway mucus transport in vivo.

Optical coherence tomography (OCT) is an imaging technique that relies on the detection of light reflection from various structures within the tissue, and moving a laser beam over the tissue generates a cross-sectional image with two-dimensional (2D) information (23). As line scanning is fast, OCT can easily reach frame rates above video rate. High-resolution OCT has successfully been used to visualize mucus transport and ciliary beating in ex vivo models (3, 6, 26).

The aim of the study was to evaluate whether microscopic optical coherence tomography (mOCT) at a resolution below 2 µm is suitable to detect and compare in vivo effects of different inhaled therapeutic interventions on mucus mobilization and clearance in *Scnn1b*-transgenic mice with muco-obstructive lung disease (27). In these mice, the expression of the β-subunit of epithelial Na^+^ channel (ENaC) is increased in epithelial cells, resulting in increased water absorption from the airway lumen, dehydration of mucus, and forming of mucus plugs (27, 42) which is mimicking the proposed hyperactivity of ENaC in CF airways (29).

We tested hypertonic saline (HS) as it was demonstrated to be an effective treatment in patients with CF and in *Scnn1b-*Tg mice and compared it to isotonic saline (IS) (11, 14, 40). On the basis of the observation that bicarbonate is reduced in the mucus layer of CF airways and that this abnormality is thought to hinder proper expansion of mucin molecules, we also assessed whether adding bicarbonate to IS improves its therapeutic efficiency (20, 25).

## MATERIALS AND METHODS

### 

#### Optical coherence microscopy system and image acquisition.

The mOCT was custom built and optimized for intravital imaging of the mouse trachea. Axial resolution at 1.25 µm inside the tissue was provided by a low noise supercontinuum light source (EXW-4 OCT; NKT Photonics, Birkerød, Denmark). A filter split box (SuperK Split, NKT Photonics) was used to guide more than 100 mW in a spectral range from 500 to 1,000 nm via a single-mode fiber (FD-7; NKT Photonics) into a Michelson type OCT interferometer. At one port of the interferometer, the output of this fiber was collimated (fiber collimator 60FC; Schäfter+Kirchhoff, Hamburg, Germany) and sent via a 50:50 beam splitter to a pair of galvanometer scanners (6210H; Cambridge Technology, Garching, Germany). A telescope consisting of two achromatic lenses (focal lengths of 50 mm and 100 mm; Thorlabs, Dachau, Germany) expanded the beam twice and imaged the mirrors of the scanner onto the back aperture of the microscope objective. Backscattered light from the tissue was recombined by the beam splitter with the reference beam, which traveled through a 10 mm block of SF57 glass for the compensation of dispersion and was reflected by a retroreflector (PS975M, Thorlabs).

The superpositioned sample and reference light was coupled via a second beam collimator into a single-mode fiber (SM600, Thorlabs), which was connected to a customized high-speed spectrometer (Thorlabs). 127,000 spectra per second covering the range from 550 nm to 950 nm were detected in 2,048 spectral channels. With 1,024 A-scans, a B-scan frame rate of 80 frames/s was achieved, including fly-back time of the galvanometric scanner. Acquired spectra were Hann-windowed and Fourier-transformed to obtain the OCT A-scans (3). Residual dispersion mismatch was numerically compensated by multiplying the complex extension of the interferogram with a correcting 9th-order polynomial phase term, which was determined from the acquired data by image optimization (21, 36, 37). Shannon’s entropy of the images was used to determine image sharpness.

#### Animals.

In all imaging experiments, either *Scnn1b-*Tg mice (27, 43) or wild-type littermates aged 4 to 6 wk were used. The mice were bred in a specific pathogen-free (SPF) facility at the University of Heidelberg and transferred to the SPF facility of the University of Lübeck at 3–4 wk of age. Animal care was provided in accordance with German law, and the study was approved by the Schleswig-Holstein state authorities (V242-7224.122-1).

#### Intravital imaging.

Mice were anesthetized by intraperitoneal injection of 500 µl of anesthesia containing 12 µg/ml fentanyl (Janssen, Neuss, Germany), 0.48 mg/ml midazolam (AlleMan Pharma, Rimbach, Germany), and 48 µg/ml medetomidine (Pfizer, Berlin, Germany) in 0.9% NaCl. Subsequently, the mice were placed on a heated intravital stage (37°C), which was adjustable in all six degrees of freedom. For imaging, the trachea was exposed by removing the overlying skin, displacing the submandibular glands, and removing the infrahyoid musculature, and the mouse was positioned to allow imaging through the intact trachea. Small tissue structures were visualized with the ×20/0.5NA objective (HCX APO L ×20/0.50 W UVI; Leica Microsystems, Wetzlar, Germany) giving a lateral resolution of 1 µm in a 1-mm field of view (FOV). Mucus transport was visualized in a larger FOV of 5 mm stretching ~5 cartilage rings down from immediately below the larynx by using the ×5/0.16 NA objective (EC Plan-Neofluar; Carl Zeiss, Oberkochen, Germany). Here, a lateral resolution of 1.8 µm was achieved. To reduce the amount of imaging data per experiment for easier data handling, we did not record continuously at 80 Hz. For quantifying mucus transport after challenge with fluid, the following imaging protocol was used.

Initially five frames at 80 frames/s (fps) were recorded every second for a time period of 10 min to determine baseline parameters. To examine the effects of therapeutic interventions, we used intranasal application that results in inhalation from a nasal depot, as it provides control over the amount of fluid given and is compatible with imaging. Furthermore, intranasal instillation of hypertonic saline was demonstrated to be therapeutically effective in *Scnn1b-*Tg mice (19). Intranasally, 30 µl of one of the following solutions were applied: 0.9% NaCl (IS), 7% NaCl (HS), or 115 mM NaHCO_3_ (bicarbonate) added to IS. The immediate effect after inhalation of these solutions was recorded by mOCT over 5 s at 80 fps. Mucus transport was then recorded with groups of five frames at 80 fps every second for 1 h.

#### Measurement of epithelial height and mucus parameters and statistical analysis.

Transport velocity of mucus was measured by manually tracking individual naturally occurring particles or inhomogeneities within the mucus using the Fiji distribution of ImageJ (35). We determined the changes in mucus layer changes due to the stimulation by measuring the distance between the highly scattering subepithelial fibers and the highly reflective air-liquid interface at four different points in the trachea at areas between the cartilage rings (illustrated in Fig. 2 and Supplemental Video S2; see https://doi.org/10.6084/m9.figshare.8863400), calculated the mean and subtracted the mean of the same measurements at baseline before the stimulation. For calculating the mucus thickness variance in each frame, the thickness was normalized by the maximum of the four values, and variance of the normalized values was determined in each frame. Epithelial height was measured by determining the height from the highly scattering subepithelial fibers to the beginning of the mucus layer, which appeared after stimulation. The epithelial height was measured in the first 10 min.

#### Statistics.

Statistical comparisons between two groups were performed by two-tailed Mann-Whitney *U* test to detect differences of treatment against control (0.9% NaCl solution). *P* < 0.05 was regarded as statistically significant.

## RESULTS

### 

#### Intravital mOCT imaging visualizes the microanatomy of the intact trachea in spontaneously breathing mice.

**Fig. 1.**
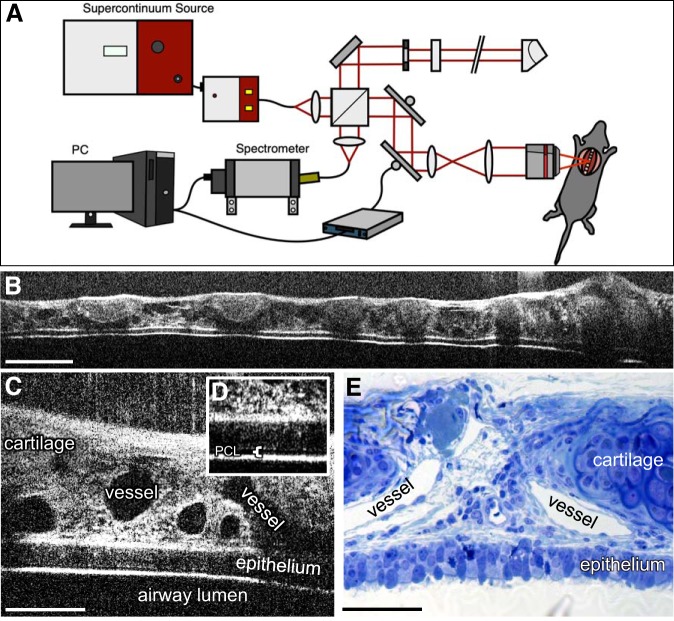
Imaging of the trachea with microscopic optical coherence tomography (mOCT) in vivo. *A*: scheme of the intravital mOCT setup. *B*–*D*: intravital imaging of mouse trachea over the length of 5 mm (*B*) and with higher magnification (*C*). The inset (*D*) shows the epithelium, the periciliary liquid (PCL), and the overlying mucus layer in higher magnification. *E*: semithin section of the murine trachea for comparison. Scale bars: 500 µm in *B* and 100 µm in *C* and *E*.

Using the newly developed mOCT setup (Fig. 1*A*), we were able to image through the entire tracheal wall of spontaneously breathing mice. This approach allowed to visualize a longitudinal section length of up to 5 mm (Fig. 1*B*). Cartilage rings and the connective tissue containing vessels, which, because of their diameter and the absence of erythrocytes are most likely lymph vessels, were readily visible (Fig. 1*C*). The tracheal epithelium was easily identifiable (Fig. 1*C*), and the interface between the mucus layer and the air appeared as a bright line. A thin layer of periciliary liquid was identifiable on top of the epithelium in some animals (Fig. 1*D*). The mOCT image gave comparable microanatomical information as a conventional histologic section (Fig. 1*E*).

#### Visualization of changes in mucus layer height and transport in wild-type and Scnn1b-Tg mice in response to inhaled isotonic saline.

**Fig. 2.**
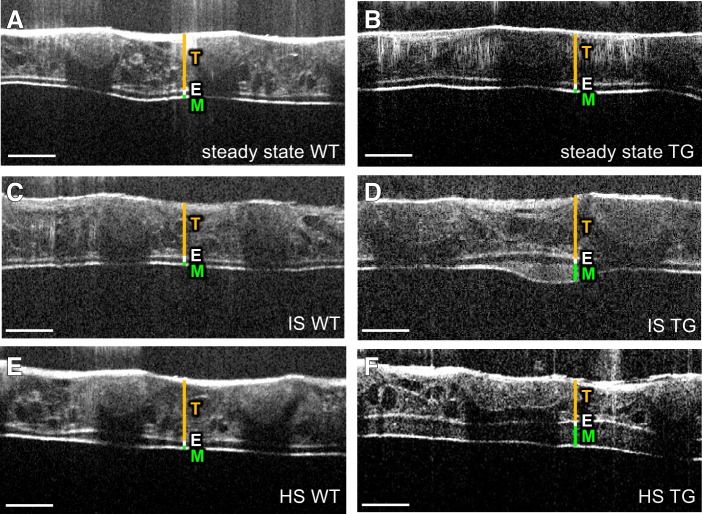
Imaging of mucus layer changes after application of isotonic saline (IS) or hypertonic saline (HS) solution. Microscopic optical coherence tomography (mOCT) images of a trachea from wild-type (WT) (*A*, *C*, *E*) and *Scnn1b*-Tg (TG) mice (*B*, *D*, *F*) under steady state (*A*, *B*), 10 min after application of IS solution (*C*, *D*), and 10 min after application of HS solution (*E*, *F*). T, tracheal wall; E, epithelium; M, mucus. All scale bars: 200 µm.

**Fig. 3.**
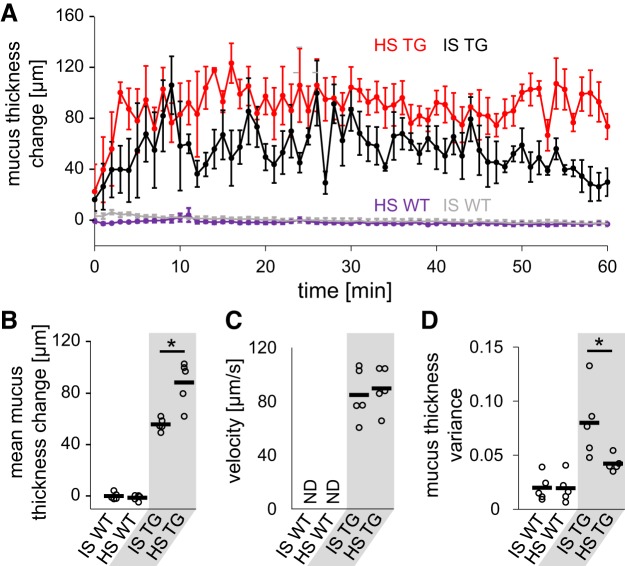
Quantitative analysis of the change of mucus layer after application of isotonic saline (IS) and hypertonic saline (HS) solution. *A*: time course of mucus thickness over time. Mean thickness for every minute ± standard error of the mean is shown. *B*–*D*: analysis of average mucus layer change over 1 h (*B*), endogenous particle velocity (*C*), and average variance of mucus layer thickness (*D*). Each dot in *B*–*D* represents data from one animal; the horizontal bar shows the mean. TG, *Scnn1b*-Tg mice; WT, wild-type mice; ND, not determined. *n* = 5 mice for each experimental condition. Statistical analysis by Mann-Whitney *U* test. *P < 0.05.

Before application of fluid, only a thin uniform layer of mucus was detected in wild-type mice, and no bulk mucus transport was observed (Fig. 2*A*). As in wild-type animals, a thin homogenous mucus layer was present in *Scnn1b-*Tg mice, and at baseline, no bulk mucus transport was detected (Fig. 2*B*). Application of IS induced transient thorax movements. Following inhalation, the mean mucus layer height increased only transiently in wild-type animals (Fig. 2*C* and Supplemental Video S1; see https://doi.org/10.6084/m9.figshare.8863394) by up to 5 µm and returned to baseline within 10 min (Fig. 3, *A* and *B*). In contrast to wild-type animals, IS application in *Scnn1b-*Tg led to an induction of bulk mucus transport (Fig. 2*D*) and an increase of mucus layer height to a mean thickness of 53 µm over the next hour (Fig. 3, *A* and *B*). Part of the mucus mobilized by IS was transported as chunks rather than as a homogenous mucus blanket (Supplemental Movie S2; see https://doi.org/10.6084/m9.figshare.8863400). By measuring the displacement of small inhomogeneities in the mucus, we determined the speed toward the larynx to be 85 µm/s (Fig. 3*C*).

#### Effects of hypertonic saline on mucus clearance in Scnn1b-Tg mice with muco-obstructive lung disease.

Intranasal application of HS in wild-type mice did not affect the mucus layer height over 1 h (Fig. 2*E*). Only a small, transient decrease of the calculated mucus layer up to 3 µm in the first minute compared with steady-state condition could be measured (Fig. 3, *A* and *B*). In *Scnn1b-*Tg mice, HS led to an increase of the mucus layer (Fig. 2*F*), with a mean thickness of 88 µm within 1 h (Fig. 3, *A* and *B* and Supplemental Video S3; see https://doi.org/10.6084/m9.figshare.8863406. By focusing on the transport rate of inhomogeneities in the mucus layer, a mucus velocity of 90 µm/s was measured, which was similar to the transport rate measured after application of IS (Fig. 3*C*). In contrast to IS treatment, inhaled HS induced a continuous mucus blanket in *Scnn1b-*Tg mice throughout the observation period after stimulation with HS (Fig. 3*A* and Supplemental Video S2; see https://doi.org/10.6084/m9.figshare.8863400, and Supplemental Video S3; see https://doi.org/10.6084/m9.figshare.8863406). To quantify and compare the changes in mucus properties after treatment, we calculated the mean spatial variance of mucus thickness, where a low mean spatial variance indicates less difference in height between individual parts of the trachea and is indicative of a more homogenous mucus blanket. Indeed, treatment with HS resulted in half of the mean spatial mucus thickness variance compared with treatment with IS (Fig. 3*D*).

#### Effects of bicarbonate on mucus clearance in Scnn1b-Tg mice with muco-obstructive lung disease.

**Fig. 4.**
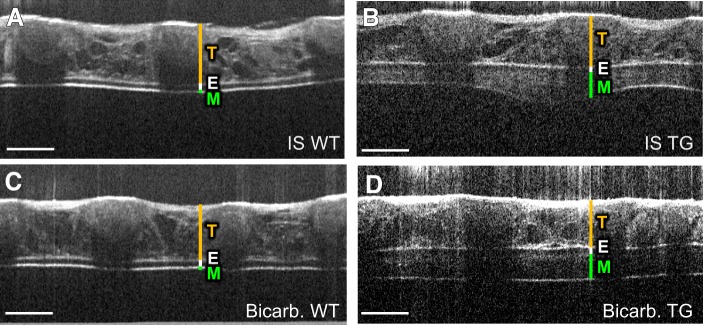
Imaging of mucus layer changes after application of isotonic saline (IS) solution supplemented with bicarbonate. Microscopic optical coherence tomography (mOCT) images of a trachea from wild-type (WT) mice (*A*, *C*) and *Scnn1b*-Tg (TG) mice (*B*, *D*) 10 min after application of IS (*A*, *B*) and 10 min after application of IS solution supplemented with bicarbonate (Bicarb.) (*C*, *D*). T, tracheal wall; E, epithelium; M, mucus. All scale bars: 200 µm.

**Fig. 5.**
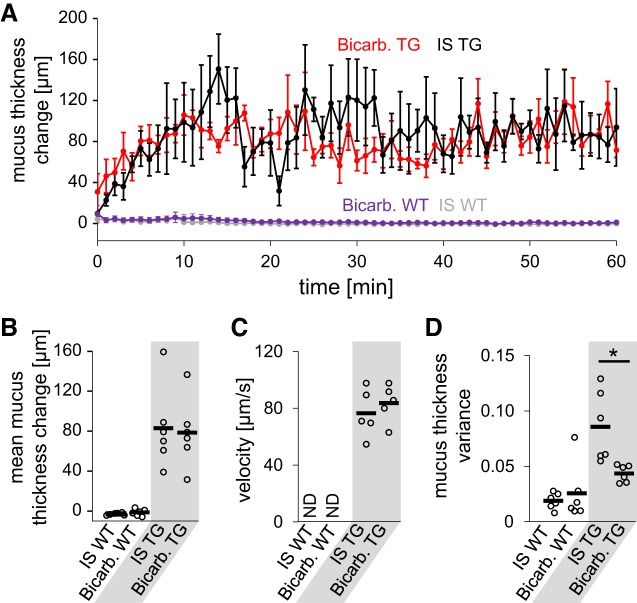
Quantitative analysis of the change of mucus layer after application of isotonic saline (IS) solution supplemented with bicarbonate. *A*: time course of mucus thickness over time. Mean thickness for every minute ± standard error of the mean is shown. *B*: analysis of average mucus layer change over 1 h (*B*), endogenous particle velocity (*C*), and average variance of mucus layer thickness (*D*). Each dot in *B*–*D* represents data from one animal; the horizontal bar shows the mean. Bicarb, bicarbonate; ND, not determined; TG, *Scnn1b*-Tg mice; WT, wild type mice. *n* = 5 or 6 mice for each experimental condition. Statistical analysis by Mann-Whitney *U* test. *P < 0.05.

Because it has been hypothesized that bicarbonate is necessary for proper unfolding of mucin molecules after secretion (1, 2, 20) and could positively influence transport of viscous mucus, we next performed additional studies to compare the effect of bicarbonate to IS. In wild-type mice, the addition of bicarbonate to IS had no effect on the height of the mucus layer (Figs. 4, *A* and *C* and 5, *A* and *B*). In *Scnn1b-*Tg mice, intranasal bicarbonate (Supplemental Video S4; see https://doi.org/10.6084/m9.figshare.8863409) increased the mucus layer by the same amount as observed for IS alone (83 µm vs. 78 µm; Fig. 4, *B* and *D* and 5, *A* and *B*), and a similar velocity of mucus transport was measured (76 µm/s vs. 83 µm/s; Fig. 5*C*). Although bicarbonate did not increase mucus amount and speed, we observed a more homogenous mucus blanket after the addition of bicarbonate and a reduced mucus thickness variance compared with IS application alone (Fig. 5*D*).

## DISCUSSION

This study shows that mOCT is able to visualize the airway microanatomy and the response to therapeutic interventions through the intact trachea in living spontaneously breathing wild-type and *Scnn1b-*Tg mice. The method was able to quantify differences between different therapeutic approaches to mobilize mucus.

Imaging of wild-type mice under baseline conditions with mOCT provided information on the microanatomy of the trachea and was able to retrieve information of the luminal airway surface without interfering with epithelial fluid balance or breathing either by directly exposing the tracheal lumen to the outside or by introduction of particles. In wild-type mice, it confirmed that the mucus layer height barely exceeds the length of the cilia. A finding supported by previous histological data obtained by perfluorocarbon-osmium fixation (27, 39).

The method also provided information on the effects of therapeutic intervention (IS, HS, IS with bicarbonate) on the mucus layer in wild-type mice. All tested interventions only very transiently changed mucus layer height. This demonstrates that healthy airway epithelium is able to rapidly absorb fluid and regulate mucus layer height, a finding also observed previously in primary airway epithelial cultures in vitro (11, 18). None of the therapeutic interventions induced bulk mucus transport in wild-type mice, which agrees with previous findings that healthy mice have a very limited ability to release mucus and do not contain mucus depots in their airways (17, 45). Although it is well described that airways from *Scnn1b-*Tg mice harbor mucus plugs (27, 42), *Scnn1b-*Tg mice did not exhibit bulk mucus transport under baseline conditions. However, inhalation of all tested solutions mobilized substantial amounts of mucus in *Scnn1b-*Tg mice that was transported toward the larynx. Our imaging approach thus confirms the presence of sticky mucus in these animals (41) that can be mobilized through therapeutic intervention.

The observation that application of IS mobilizes mucus in *Scnn1b-*Tg mice is consistent with the concept that improved hydration of dehydrated/hyperconcentrated mucus reduces mucus viscosity and increases its transportability (8, 28). In CF patients, HS was superior in mobilizing mucus compared with IS (15, 33, 34). In agreement with these data, we observed that HS mobilized more mucus compared with IS in *Scnn1b-*Tg mice, as a thicker layer was transported. Notably, the amount of mobilized mucus was not the only difference observed. After application of HS, the mucus was more homogenously distributed over the epithelium in a blanket-like fashion, as compared with IS that mobilized mucus chunks. This observation together with the ability of HS to increase mucus amount compared with IS supports the hypothesis that the increased osmolarity of HS draws more water into the airway lumen, which subsequently better hydrates mucus compared with IS, leading to a higher mucus volume and more homogeneous, less viscous, mucus (18). However, our data do not rule out that part of the increased amount of mucus is due to HS-induced mucus secretion (4, 5) or direct release of mucus tethered to epithelial cells (16).

The addition of bicarbonate to IS did not increase the mucus amount compared with IS alone but changed the properties of mucus comparable to HS in *Scnn1b-*Tg mice. The ability of bicarbonate solution to change mucus properties without increasing mucus volume points toward a different mode of action for bicarbonate compared with HS. Adding bicarbonate to IS only moderately increased hypertonicity from 308 mosmol/L to 538 mosmol/L compared with the 2396 mosmol/L of the HS solution used. Therefore, a purely osmotic effect of bicarbonate addition is unlikely. Our observations favor the concept of a direct effect of bicarbonate on mucus properties (38). Indeed, shielding of calcium ions that otherwise prevent the proper unfolding of mucins has been proposed as the main function of bicarbonate in CF airways (31). It is worthy to note that *Scnn1b-*Tg mice do suffer from dehydration of mucus rather than bicarbonate deficiency in the mucus layer, which was described in other CF-models, based on genetic knockout of CFTR (20, 22). The ability of bicarbonate to alter mucus properties in *Scnn1b-*Tg mice indicates that its mode of action is not restricted to mucus deficient in bicarbonate.

There are two major limitations of our approach. First, under basal conditions, the mucus layer was too homogenous and did not provide enough inhomogeneities to determine mucus transport at baseline. Measuring this parameter would require introduction of contrast-enhancing substances such as particles that could alter baseline transport. A second limitation is the inability to monitor individual lower airways of the mouse to directly visualize the effect of therapy on distal mucus plugs. Direct visualization of smaller airways would require surgical opening of the thorax wall to expose smaller airways, which is incompatible with spontaneous breathing. It should be noted, however, that imaging through the intact trachea has the advantage that it integrates information on all mucus plugs that were mobilized rather than focusing on individual mucus plugs that might show more variability with respect to treatment response. An alternative approach would be endoscopic imaging, which is not feasible in smaller animals due to the small diameter of the trachea that would require a probe small enough not to impair breathing, which is currently not available. It should also be noted that even small probes pose the risk of interfering with mucus transport in larger species.

Our data demonstrate that mOCT imaging through the intact trachea is suitable to visualize the microanatomy of the trachea, including the epithelium and its mucus content. It allows to monitor the effects of mucus-mobilizing therapies and is sensitive enough to quantify differences between individual therapeutic approaches. We further conclude that mOCT is a promising tool for further preclinical characterization of drugs that are under development to improve mucus properties and clearance in muco-obstructive lung diseases such as CF and COPD.

## GRANTS

The study was supported by the German Ministry for Education and Research (82DZL004A1 and 82DZL001A2). M. A. Mall is supported by a grant from the Einstein Foundation Berlin (EP-2017-393).

## DISCLOSURES

No conflicts of interest, financial or otherwise, are declared by the authors.

## AUTHOR CONTRIBUTIONS

M.P., H.S.-H., M.A.M., G.H., and P.K. conceived and designed research; M.P. performed experiments; M.P., H.S.-H., M.A.M., G.H., and P.K. analyzed data; M.P., H.S.-H., M.A.M., G.H., and P.K. interpreted results of experiments; M.P. prepared figures; M.P., H.S.-H., and P.K. drafted manuscript; M.A.M. and G.H. edited and revised manuscript; M.P., H.S.-H., M.A.M., G.H., and P.K. approved final version of manuscript.
